# Justice enablers of climate-health adaptation in South America

**DOI:** 10.1016/j.joclim.2025.100459

**Published:** 2025-06-13

**Authors:** Romina Rekers, María Victoria Gerbaldo, Carlos Yabar, Cintia Rodríguez Garat, Lucas Rekers

**Affiliations:** a Department of Philosophy, University of Graz, Austria; b National University of Cordoba, Argentina; c National Institute of Health of Peru, San Martín de Porres University, National University of Trujillo, Peru; d National University of La Plata, Argentina; e Tres de Febrero University, Argentina

**Keywords:** Climate adaptation, Health, Justice, South America

## Abstract

**Background:**

Integrating justice enablers into climate-health adaptation planning reduces failed adaptation, prevents maladaptation, and facilitates transformative change in health systems. This is particularly necessary in South America (SA), where climate adaptation research and policy are financially constrained. By carefully considering differentiated climate-health risks and necessary trade-offs, National Adaptation Plans (NAPs) and Health National Adaptation Plans (HNAPs) provide forums for identifying just adaptation paths. This study assesses the integration of recognitional, procedural, and distributive justice enablers in climate-health planning in SA.

**Methods:**

Justice enablers were tracked within the actionable sections of the NAPs, HNAPs, or their Subsidiary Adaptation Strategies (SASs) of the South American countries. The level of integration of justice enablers was measured using keywords from the climate adaptation literature.

**Results:**

There is a significant disparity among countries in the level of integration of justice enablers in national adaptation planning. There is also significant variation across countries in the integration of different dimensions of justice (recognitional, procedural, distributive). Most countries score higher on the integration of recognition justice than on procedural and distributive justice.

**Conclusion:**

Comprehensive frameworks are required to integrate justice considerations into climate-health adaptation planning in a way that avoids failed adaptation or maladaptation.

## 1 Introduction

Climate-health adaptation in South America (SA) is urgent. This is due to the low level of resilience of health systems, overlapping vulnerabilities within the population, and related exposure pathways associated with precarious livelihoods [1]. Local populations are widely overexposed to social and environmental determinants of poor physical [2] and mental health [3]. In response to this scenario, which is common to regions of the Global South, the World Health Organisation (WHO) plans to develop a global action plan on climate change and health by 2025 [4]. COP28 has also urged parties to the Paris Agreement to develop country-led, gender-responsive, participatory, and fully transparent National Adaptation Plans (NAPs) by 2030 [5].

The ability to reduce the risk of climate change to the health of the SA population [6] varies considerably depending on local constraints [7], such as financial constraints, and enabling conditions such as participatory adaptation strategies [8]. Under these conditions, governments need to maximise adaptation opportunities by improving their institutional frameworks [9]. National Adaptation Plans (NAPs) and Health National Adaptation Plans (HNAPs) are instruments that fulfil this role, for example, by increasing access to climate-change funding from key climate funding streams [10].

NAPs and HNAPs also play an important role in the climate transition. Reducing the risks of climate change to health requires a transition towards climate resilience development [11], and adaptation planning is one of the pillars of this transition [12]. Transitions involve two types of tasks. First, it requires identifying *transitional pathways*, that is, finding out ‘what works’. Nevertheless, following any transition path triggers conflicts of interests, expectations, and rights trade-off. To adjudicate these conflicts, a second task is necessary, which is to identify *just pathways*. In this context, NAPs and HNAPs provide national fora for identifying these just pathways by carefully considering differentiated climate-health risks and necessary trade-offs, and by including different views [13]. Assessment of enabling conditions in adaptation planning has made use of justice-embedded indicators but has been focused on ‘what works’ and the extent to which countries have progressed along these pathways [14]. However, adaptation planning comes with many justice challenges. Failure to address these normative aspects can lead to failed or unjust transitions.

Opportunities for successful climate-health adaptation can be maximised by including justice enablers, i.e. considerations that increase the chances of successful adaptation, in NAPs and HNAPs [[15], [16]]. This study sets out to assess the integration of recognitional, procedural, and distributive justice enablers in climate-health planning in SA. While there may be differing interpretations of these dimensions of justice, we have opted to use conceptions referenced in the literature on climate adaptation [17].

*Recognitional* justice, as we understand it, refers to the inclusion and recognition of legitimate actors’ agency and the acknowledgement of their rights, needs, and interests [17]. In climate adaptation planning, considerations of recognitional justice identify marginalised populations, assess the impacts of climate change on them, and take into account existing injustices that already affect these groups [18]. *Procedural* justice refers to the fairness of political decision-making by including the relevant stakeholders [19]. Procedural justice enablers usually consist of participation mechanisms that aim to correct power asymmetries that increase climate vulnerability [17]. The *distributional* dimension of justice is a forward-looking aspect of justice concerned with allocating scarce resources [20]. Distributive justice enablers aim to prevent existing inequalities from being reproduced or exacerbated [17].

*Justice enablers* create conditions that promote successful adaptation by recognising the differential vulnerability of some groups to climate impacts on health. They also deal with the interests, expectations, and rights of those affected by climate-health policies, involving them in the decision-making process and assessing different distributive outcomes. The integration of justice enablers also reduces the likelihood of failed adaptation by removing some feasibility constraints. For example, the implementation of just procedures for decision-making has the capacity to reduce social conflict [16]. Justice enablers also pre-empt maladaptation, unjustified trade-offs in adaptation policy that exacerbate or shift vulnerability or exposure [17]. Incorporating justice enablers also promotes the creation of conditions for transformative change, a kind of change that revises the goals and values of systems [11]. For example, recognising Indigenous knowledge systems and integrating them through participatory mechanisms has the potential to transform the functions of the health system by adopting a more holistic approach to health that goes beyond the mere adaptation of existing practices [21].

There are many examples of how incorporating justice enablers into climate-health adaptation planning has contributed to successful adaptation [17]. In Colombia, for instance, a gender-responsive approach to climate adaptation has been implemented to address the disproportionate impacts of climate change on women and marginalised groups. Initiatives have focused on integrating women’s voices into climate decision-making processes, recognising that women often possess critical knowledge about local ecosystems and resource management. These efforts have contributed to a more equitable distribution of resources and benefits from adaptation actions, enhancing resilience among vulnerable populations [18].

This study assesses the integration of recognitional, procedural, and distributive justice enablers in climate-health planning in SA. We posited that the incorporation of justice enablers contributes to the construction of representations and the shaping of narratives around climate-health adaptation policies. This is so because, when words are incorporated into states’ commitments, they trigger several consequences. Words allow people to compare their resources and contribute to the development of positional goods [22]. The inclusion of justice enablers in state commitments generates expectations that, if consistent with considerations of justice, are legitimate and therefore influence the design of our life plans and carry moral weight in the design of climate policy [23].

## 2 Method

This study is an exploratory corpus analysis of NAPs, HNAPs, or their SASs (Subsidiary Adaptation Strategies) from the perspective of justice enablers of climate-health adaptation. Although the focus of this study is climate-health adaptation, we also incorporated NAPs into the analysis, since multi-sectoral collaborations across national agencies are key to preventing health adaptation from becoming siloed [11]. Additionally, many countries include climate-health adaptation plans within their NAPs, especially when they have not advanced towards sector-based planning. Recognising that countries and sectors progress at their own pace and adopt different approaches [16], SASs were also incorporated when no NAPs or HNAPs were found. SASs include a national climate strategy with climate adaptation among their goals and national health plans with climate change as one of the pillars.

The study sample included nine NAPs, four HNAPs, and 13 SASs from 12 South American countries: Argentina, Bolivia, Brazil, Colombia, Chile, Ecuador, Guyana, Paraguay, Peru, Uruguay, Venezuela, and Suriname. French Guiana was not included in the sample as it was an overseas department of France. The NAP submitted by Uruguay to the UNCCC portal was considered a sectoral NAP on agriculture. Although Uruguay had no NAP, the sectoral NAPs for agriculture, coastal zones, and cities and infrastructure were considered its SAS since Uruguay adopted a multisectoral approach to develop its national adaptation policy.

Justice enablers were tracked within the actionable parts of the NAPs, HNAPs, or their SASs. These parts included adaptation goals, strategies and actions, financial aspects, and monitoring and evaluation strategies. Importantly, the diagnostic sections of the documents were not included. A broad interpretation was applied in selecting the actionable part of each plan. Where there was doubt as to the actionable nature of a section, a re-analysis was carried out. A set of keywords for each dimension of justice was identified in the adaptation literature and tracked in the documents (Table 1, supplemental material) [17]. The keyword selection and their categorization under each dimension of justice draw on the underlying sectoral and regional chapters, as well as a synthesis of adaptation literature from the IPCC 6AR [15]. This categorisation needs to be improved in future research, as it reflects the lack of a comprehensive approach to justice considerations for climate research and policy [19]. The accuracy of the keyword analysis was checked against a bottom-up analysis of the Uruguayan NAP, which showed that the selected keywords excluded only insignificant cases of justice considerations.

The keywords were translated into Spanish. The keywords were not translated into Portuguese, as the Brazil NAP and HNAP were written in English (S1). To reflect the linguistic diversity of SA, we also incorporated terms that may not have been literal translations, but conveyed the same idea based on local usage. For example, in contrast to the English language literature, the term “vulnerable communities” was more commonly used locally than vulnerable groups. Justice enablers were collected after ruling out irrelevant incidents (e.g., those that used ‘just’ for ‘only’) (S2).

The level of integration of justice enablers was measured in 72 cases. Each case represents a different dimension of justice (recognitional, procedural, distributive) in the NAPs, HNAPs, and SASs of each country. The enabling potential of each case was then classified as low, medium, or high depending on its position relative to the threshold marked by the leading case, the case with the highest integration of justice enablers. Two additional thresholds were set forth to measure the distance from the leading case, as shown in Table 3, supplemental material.

## 3 Results

### 3.1 Disparities in climate-health adaptation planning

Despite the urgency of adaptation, more than a decade after the process to formulate and implement NAPs was established [6], only 52 countries have submitted their own [24]. In SA, there appears to be a variation in the extent to which countries have progressed in their adaptation planning efforts. Only eight countries have developed their NAPs: Argentina, Brazil, Chile, Colombia, Ecuador, Paraguay, Peru, and Suriname. Chile is currently in the process of revising its NAP, while Argentina’s and Ecuador’s NAPs are not listed on the UNFCCC portal. Bolivia, Guyana, and Venezuela have not yet developed their own NAPs (Table 2, supplemental material). It is also worth noting that the existing plans face significant limitations, including a lack of funds allocation, involvement from health-related institutions, and measurable indicators to guide policymakers [26].

Assessments of the level of engagement with health have shown that all NAPs highlight health as a high-priority sector [26]. Until 2015, however, the health sector was only included in the NAPs of three SA countries, and by 2020, Brazil was the only country in the region that reported conducting a climate-health vulnerability and adaptation assessment, which did not influence the allocation of human or financial resources [27].

Although health is one of the thematic areas of many NAPs [16], HNAPs are equally important because climate impacts differ by sector, and frequently, budget is allocated by area [16]. A sector-based approach has the potential to enhance the operational and feasibility levels of adaptation plans by engaging stakeholders from various sectors. Thus, the importance of HNAPs must be acknowledged as essential for identifying actions to tackle climate impacts on health and for developing robust strategies for specific climate-related health emergencies [25]. HNAPs can also address the lack of participation of health actors in climate policy and build capacity for them to participate in national adaptation planning [11].

Despite the important role of HNAPs in addressing the adaptation gap [28], only four countries in SA have developed them—Brazil (2016), Chile (2017), Suriname (2019), and Argentina (2023). Moreover, only two countries HNAPs —Brazil and Suriname— are listed on the portal. Although Uruguay is developing its HNAP, neither Uruguay nor Venezuela currently has a SAS (Table 2, supplemental material).

### 3.2 Recognitional justice

The recognitional dimension of justice is concerned with the question of what historical, cultural, and regional factors climate policy should be sensitive to [26]. The lack of recognition of the interests of local communities, for example in the governance systems of carbon sinks such as forests, leads to maladaptation strategies [29].

**Fig. 1:**
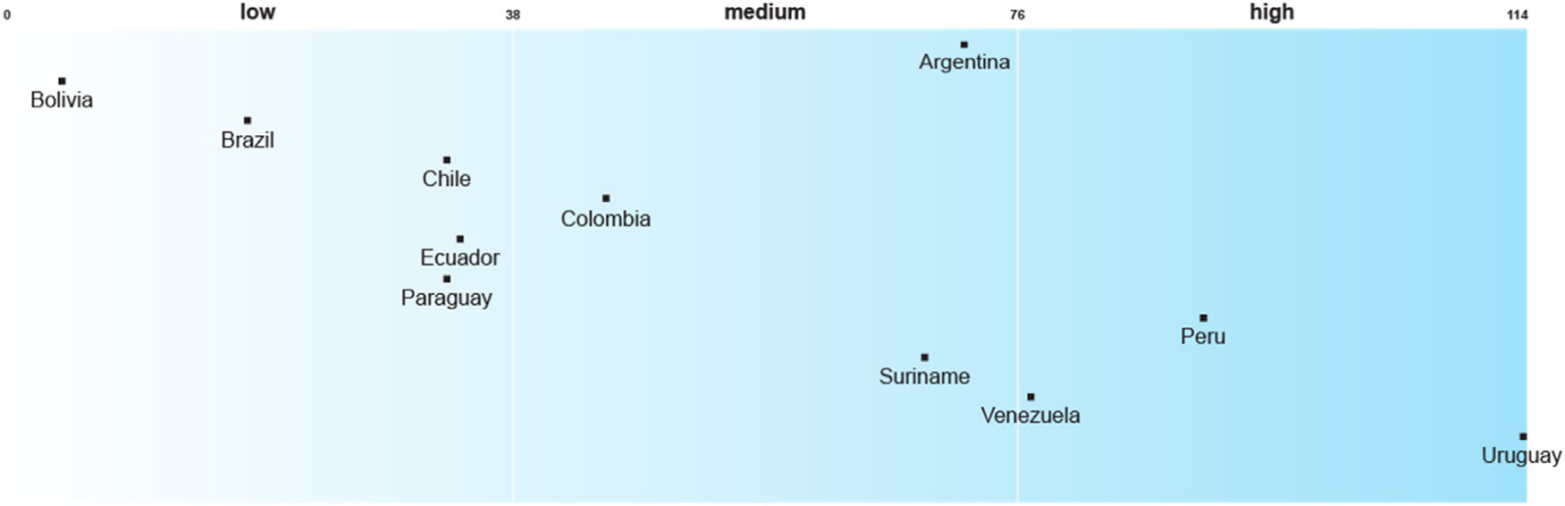
Recognitional justice enablers in National Adaptation Plans or their Subsidiary Adaptation Strategies Not applicable: Guayana.

Uruguay is at the forefront of integrating recognitional justice enablers into its national adaptation planning in the SA region. Most of the other countries rank low in this respect, having included few justice enablers into their NAPs or subsidiary strategies (Fig. 1). Among the high-scoring countries, Peru’s NAP stands out for the integration of recognitional justice strategies that include gender, interculturality and intergenerational relations as transversal dimensions. The gender perspective is presented as an analytical and methodological category with policy implications. At the same time, its intercultural approach recognises cultural differences as one of the pillars of a democratic climate policy. The Peruvian NAP incorporates different conceptions of well-being and development from different ethnic-cultural groups. It seeks complementarity between traditional and scientific knowledge, recognising ancestral practices and knowledge as well as local perceptions and goals, such as development in harmony with nature. It also appoints and plans training for women leaders to preserve traditional knowledge.

**Fig. 2:**
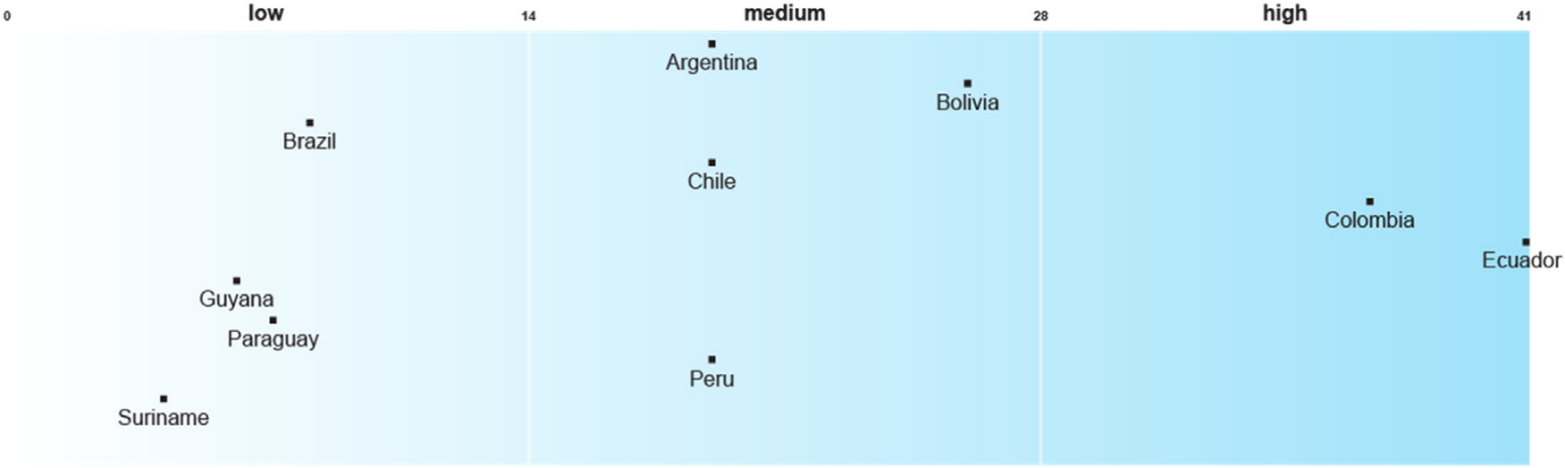
Recognitional justice enablers in Health National Adaptation Plans or their Subsidiary Adaptation Strategies Not applicable: Uruguay, Venezuela.

Despite the importance of recognition in climate-health planning in unstable contexts [30], the HNAPs in SA contain significantly fewer recognitional justice enablers than the NAPs. Ecuador leads in the integration of recognitional justice considerations, followed by Colombia in second place. The remaining countries are evenly distributed between low and medium scores (Fig. 2).

Some recognitional enablers stand out in the Ecuador and Colombian national strategies. Ecuador’s ten-year health plan includes the concept of ‘double vulnerability’ to develop an intersectoral social protection strategy on health determinants, such as the nutritional gap between Indigenous and non-indigenous children. It also includes victims of gender-based violence and child abuse among the priority groups. Colombia’s ten-year health plan adopts an intersectoral and cross-sectoral approach to identifying population and territorial differences in climate-health risks and adaptation needs. It also articulates integral networks such as the Intercultural Indigenous Health System.

### 3.3 Procedural justice

Procedural justice contributes to the legitimacy of climate-health policies and increases the acceptability of decision-making outcomes by ensuring that agreed procedural rules are followed [18]. Considerations of procedural justice that guide adaptation planning include involvement in plan design and participation in monitoring and evaluation [19].

**Fig. 3:**
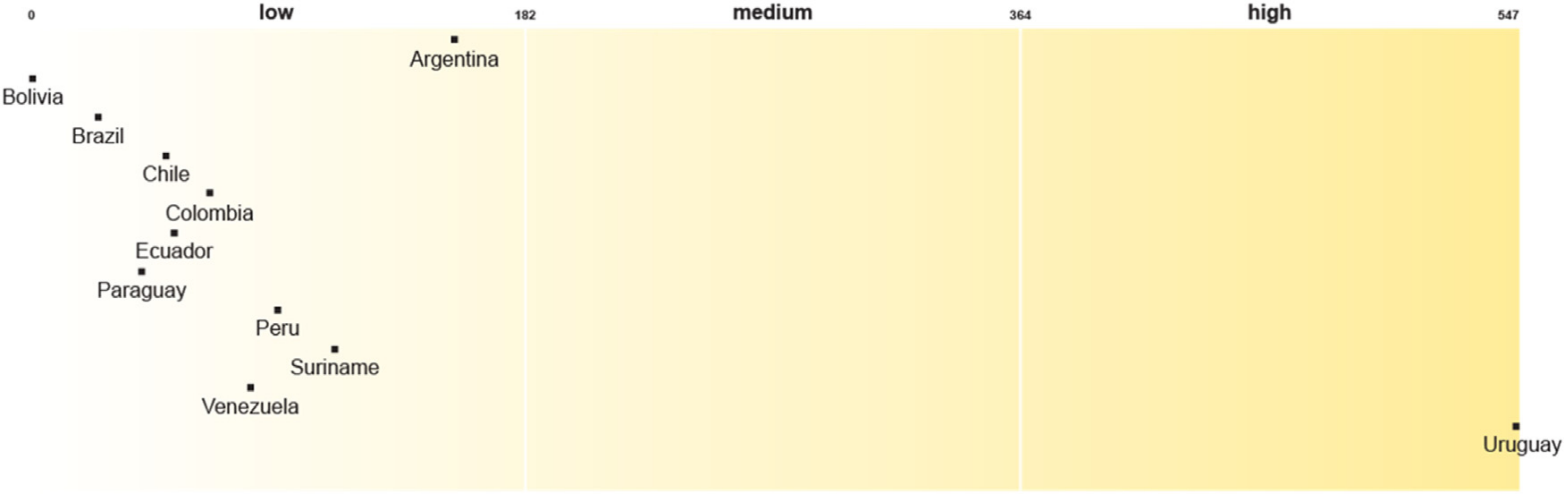
Procedural justice enablers in National Adaptation Plans or their Subsidiary Adaptation Strategies Not applicable: Guayana.

Compared to the integration of recognitional justice, there is a greater degree of asymmetry among countries in the integration of procedural justice. Most countries´ NAPs score low, with some outliers (Fig. 3). Uruguay is a regional leader in the integration of procedural enablers, however, the participation of stakeholders, including those who bear the costs of adaptation policies, is underdeveloped in a way that can lead to maladaptation. Its national plan for the agricultural sector identifies the ‘beneficiaries’ of adaptation measures, such as farmers. However, it does not identify the ‘losers’ of adaptation, those who may bear the costs of adaptation policy. Moreover, stakeholder participation is limited to both the development and adjustment of adaptation measures, while the implementation, monitoring, and evaluation phases are mainly confined to the inter-institutional sphere. In this way, procedural justice commitments are limited to some restricted tasks and roles.

**Fig. 4:**
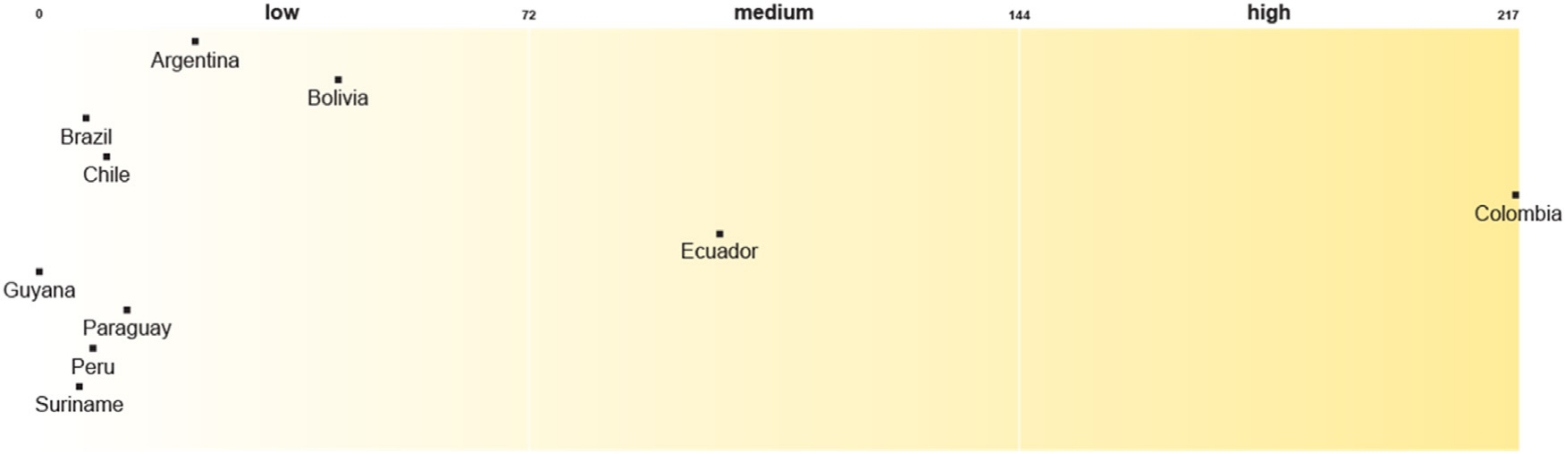
Procedural justice enablers in Health National Adaptation Plans or their Subsidiary Adaptation Strategies Not applicable: Uruguay, Venezuela.

The asymmetry in the integration of procedural enablers is also reflected in the HNAPs or their SASs. Colombia leads the region in the integration of procedural justice considerations and sets the threshold. While Ecuador is in the middle, the remaining countries are in the group with lower levels of integration of procedural justice enablers (Fig. 4). Although Colombia’s ten-year health plan is based on intersectoral action, which contributes to its procedural legitimacy, it does not specify the actions and roles of stakeholders. Even though Ecuador’s ten-year health plan acknowledges the different *pueblos* and nationalities, what is necessary condition for their inclusion in decision making process, the identification of non-institutional stakeholders remains vague.

### 3.4 Distributive justice

Distributive justice enablers aim to ensure that the benefits and burdens of adaptation policies are fairly distributed [3]. Distributive justice considerations in adaptation planning have focused on health, safety, buildings, professional development opportunities, green infrastructure, food and transport [19]. Currently, climate adaptation assessment models do not take into account distributive outcomes [30], reducing the likelihood that national adaptation planning will address them.

**Fig. 5:**
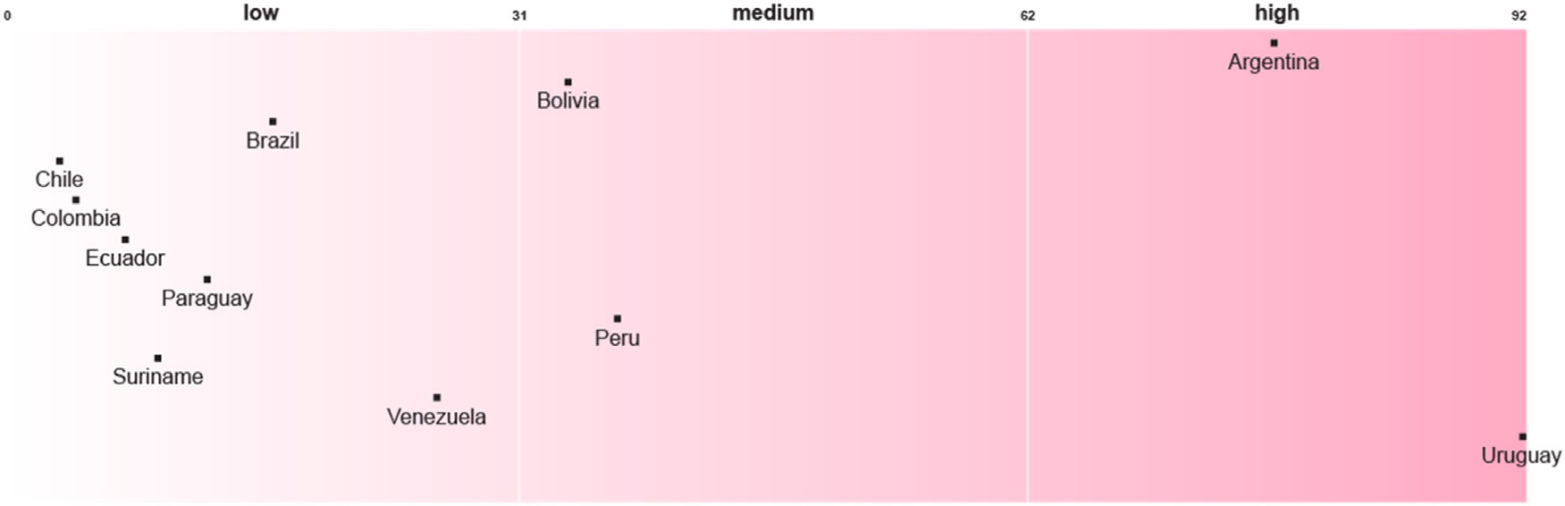
Distributive justice enablers in National Adaptation Plans or their Subsidiary Adaptation Strategies Not applicable: Guayana.

Uruguay’s NAP is the leader in the integration of distributive justice enablers, followed by Argentina, Bolivia, and Peru with medium scores, and the remaining countries with low scores (Fig. 5). Uruguay’s NAP makes a strong commitment to the financial component of meeting climate change adaptation needs, with a particular focus on women and children. However, the challenges of justice are only superficially addressed and the pathways of adaptation towards just outcomes remain vague.

The inclusion of vulnerable communities and funding for adaptation of poor and vulnerable social groups are key distributive enablers in Argentina’s and Peru’s NAPs. Argentinian NAP stands out for adopting a gender perspective in updating the Social Vulnerability Index and for identifying specific measures to strengthen the resilience of women and the LGBTQ+ community. However, its content is not translated into concrete measures and is thus limited to setting aspirational targets.

Distributive considerations related to women are the most frequently and consistently addressed enablers in the NAPs of Argentina, Uruguay, and Peru. The Peruvian NAP integrates distributive enablers with a gender focus on women, girls, and adolescents. It also addresses gender identity and equity in care. It includes supporting measures and a financing plan focusing on families living in poverty or extreme poverty.

**Fig. 6:**
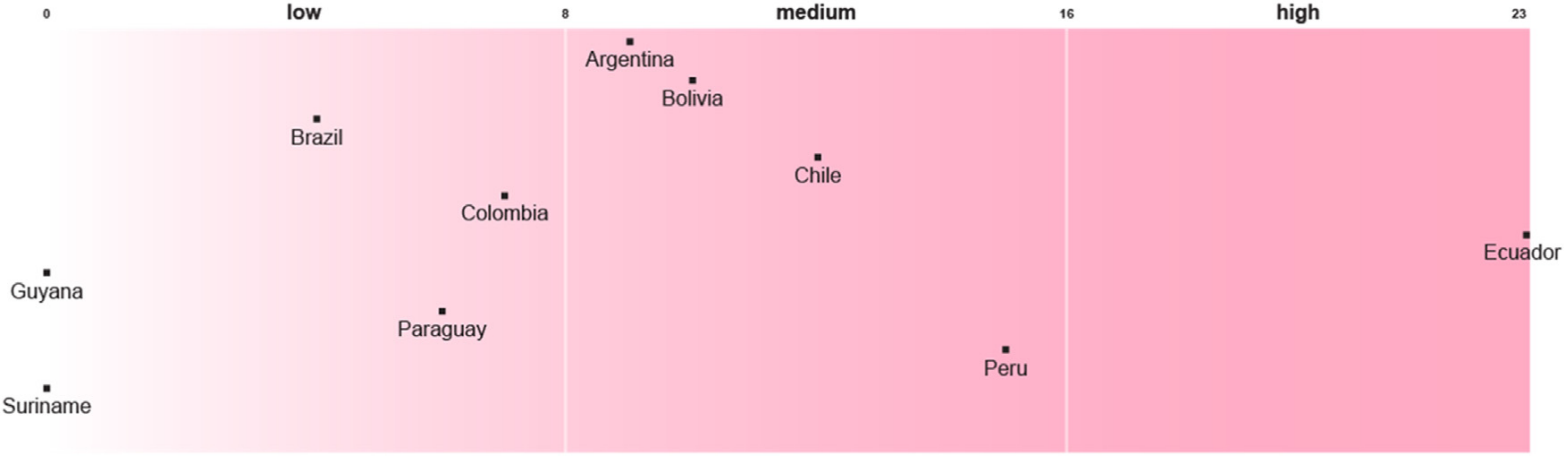
Distributive justice enablers in Health National Adaptation Plans or their Subsidiary Adaptation Strategies Not applicable: Uruguay, Venezuela.

In terms of HNAPs or their SASs, Ecuador leads the group while the other countries are almost equally divided between medium and low scoring groups (Fig. 6). Ecuador has prioritised vulnerable groups such as the LGBTQ+ community and sex workers. It sets out an intersectoral social protection strategy to reduce inequalities and disparities, which includes promotion, prevention and education to empower the most vulnerable communities, mainly women exposed to natural disasters. Ecuador and some countries with a medium score developed strategies for climate-health emergencies and integrated intra- and intergenerational justice considerations into budget planning.

## 4 Discussion

NAPs and HNAPs are best understood as a continuous process that starts with diagnosing problems, identifying stakeholders and priorities, and developing an action plan. This process continues with monitoring, evaluation, and learning, and then starts over again. In this context of this continuous process, the integration of justice enablers shapes adaptation narratives, representations, and contestation, and provides indicators that feed into the different stages of a continuous and intergenerational process.

National adaptation planning shows important disparities in the integration of different dimensions of justice (Table 3, supplemental material). In both NAPs and HNAPs, most countries score higher in the integration of recognitional justice than procedural and distributive justice. Procedural and distributive justice show greater asymmetry among countries. These asymmetries may be related to the fact that most climate-health research has focused on the impacts of climate change on population health and health burdens, while less attention has been paid to transformative strategies [31]. Giving more space to questions of burden sharing and benefits of adaptation allocation may facilitate the identification and recognition of relevant stakeholders. Methods and principles for allocating adaptation benefits and burdens have yet to be developed. The fact that there is no universal metric to assess the wide range of adaptation outcomes and that adaptation is poorly represented in quantitative models used in climate research [32] may also influence the lack of consideration of distributive justice in NAPs and HNAPs.

The fact that most countries fall short in integrating distributive enablers is of concern, as this may promote climate-health policies with unaccounted burdens or shifts in vulnerability, leading to failed adaptation and maladaptation [3]. The lack of distributive considerations in adaptation planning also prevents the development and implementation of transparent and coherent guidelines for addressing allocation dilemmas and social conflicts related to resource scarcity during extreme weather events and public health emergencies.

While it is common to consider transitional losers in mitigation scenarios, those who bear the burdens of transition [33], identifying them is a pending task for adaptation research and policy. As adaptation policies become more necessary due to growing climate-health risks, the conflict of interests, expectations, and rights increases. In addressing these conflicts, populations will seek to have a fair influence on climate policies, adopting, among other strategies, the justice heuristic provided by the NAPs and HNAPs. In this context, a high level of integration of procedural and distributive justice enablers in adaptation planning is a matter of practical necessity. On the other hand, the identification of adaptation burdens and transitional losers is likely to generate new procedural and distributive debates that will inform future NAPs and HNAPs. Opportunities to address these issues in the near future include Chile’s NAP under reform and Uruguay’s HNAP ongoing process (Table 2, supplemental material).

Integrating justice enablers of adaptation in NAPs and HNAPs is also necessary for assessing how much governments are doing to protect the human right to health of their residents. The human rights obligations of national governments in climate emergencies are the subject of ongoing advisory proceedings at the Inter-American Court of Human Rights [34]. In assessing the degree of progress and non-regression in the protection of the human right to health [33], NAPs and HNAPs complement the national climate legal frameworks and regional regulations such as the Escazú Agreement on access to information, public participation, and environmental justice [35].

## 5 Limitations

This seminal study on justice enablers in climate-health adaptation planning reveals the need for a normative framework that distinguishes between dimensions (recognitional, procedural, distributive, intergenerational, transitional) and conceptions of justice (liberal, communitarian, etc.) Adaptation planning in SA shows significant variation in the integration of these justice enablers. Comprehensive frameworks still require further development. Completing this pending task is key to achieving a just transition, preventing failure and maladaptation, and promoting the kind of change that transforms the paradigms, goals, and values structuring health systems.

Other important dimensions of justice, including transitional, intergenerational, corrective, and epistemic justice, were not included in this study. They should be a subject of future studies. For example, although epistemic justice is an important prerequisite for successful adaptation, it has not been considered an independent justice enabler in the adaptation literature [3]. Epistemic justice can be seen as a form of recognitional justice [36], a prerequisite for procedural justice [38]. It is also associated with inequitable distribution of valuable epistemic goods [37]. Epistemic justice considerations in NAPs and HNAPs can be traced indirectly through enablers such as local knowledge, traditional knowledge, knowledge systems, Indigenous knowledge and knowledge co-production.

In developing this study, we identified a practice that can be considered a case of epistemic injustice. We have termed this practice ‘epistemic washing’ [39], which occurs when adaptation planning includes stakeholder contributions but it does not show how their inputs have influenced the outcome of the process [34].

NAPs and HNAPs involve an intergenerational process that contributes to building intergenerational climate resilience [39]. Although the temporal dimension of adaptation options is understudied in the literature [40], progress has been made in SA in recognising the interests of future generations at the legal and policy levels. In this direction, the Inter-American Human Rights System recognises that climate change constitutes an emergency for the human rights of future generations [40]. On the other hand, some NAPs — Argentina, Chile, Paraguay, Peru, Uruguay — and HNAPs or their SAS — Ecuador, Venezuela — adopt an intergenerational approach. However, they still lack concrete strategies for taking into account the interests of future generations in adaptation research and policy.

## Funding

This research was made possible by an award from the Oxford-Johns Hopkins Global Infectious Disease Ethics Collaborative (GLIDE), which is funded by Wellcome (320225) and the Austrian Science Fund (FWF) project T 1323-G.

## CRediT authorship contribution statement

**Romina Rekers:** Writing – review & editing, Writing – original draft, Supervision, Project administration, Methodology, Investigation, Funding acquisition, Formal analysis, Conceptualization. **María Victoria Gerbaldo:** Writing – original draft, Methodology, Investigation, Conceptualization. **Carlos Yabar:** Writing – review & editing, Writing – original draft, Methodology, Investigation. **Cintia Rodríguez Garat:** Writing – original draft, Methodology, Investigation. **Lucas Rekers:** Writing – review & editing, Writing – original draft, Methodology, Investigation, Conceptualization.

## Declaration of competing interest

The authors declare that they have no known competing financial interests or personal relationships that could have appeared to influence the work reported in this paper.

## Appendix Supplementary materials

Image, application 1

## Footnotes

Supplementary material associated with this article can be found in the online version at doi:10.1016/j.joclim.2025.100459.
