# Supplementary material for: Justice enablers of climate-health adaptation in South America
Source: J Clim Chang Health. 2025 Jun 13;23:100459. doi: 10.1016/j.joclim.2025.100459 (PMC12851179; doi:10.1016/j.joclim.2025.100459)
Supplement: Supplementary file 1 [file mmc1.docx]

**Data in brief: Justice enablers of climate-health adaptation in South America Abstract**

This dataset provides a detailed analysis of justice enablers - recognition, procedural and distributive justice - in climate-health adaptation planning in South America. It includes data from National Adaptation Plans (NAPs), Health National Adaptation Plans (HNAPs) and Subsidiary Adaptation Strategies (SASs) from 12 South American countries. The dataset tracks the integration of equity considerations using keyword-based analysis and classifies the data as having low, medium or high potential to enable successful climate adaptation. This dataset is a valuable resource for researchers and policy-makers studying equity and justice in climate and health adaptation policies.

**Specifications table**

| **Field** | **Description** |
| --- | --- |
| **Data format** | Tables, Figures, CSV, Excel, Text document, raw files. |
| **Experimental factors** | Recognitional justice, procedural justice, distributive justice |
| **Data source location** | South America (12 countries: Argentina, Bolivia, Brazil, Chile, Colombia, Ecuador, Guyana, Paraguay, Peru, Suriname, Uruguay, Venezuela) |
| **Time period** | Data collected from NAPs, HNAPs, and SASs published up to 2023 |

**Value of the Data**

- This dataset provides a comprehensive overview of justice enablers in climate-health adaptation planning across South America.
- Researchers can use this dataset to analyze disparities in the integration of recognitional, procedural, and distributive justice in national adaptation plans and health national adaptation plans or their subsidiary adaptation strategies.
- Policy makers can use this data to identify gaps in justice considerations and develop more equitable adaptation strategies.
- The dataset supports comparative studies on climate adaptation policies across different regions and countries.

**Data Description:**

**Table 1**

Justice enablers

| **Recognitional justice** | **Justicia de reconocimiento** |
| --- | --- |
| plural, plurality, vulnerable, knowledge systems, indigenous knowledge, local knowledge, traditional knowledge, recognition, inclusion, agency, acknowledged, historical inequalities, structural inequalities/regional inequalities, distribution of adaptation benefits, poor, poorest, marginalized, equitable adaptation priorities, adaptation priorities, marginalized groups, co-production of knowledge. | plural, plural, pluralidad, vulnerable, vulnerabilidad, grupos, comunidades, poblaciones vulnerables, estratos socioeconómicos más bajos, situación de pobreza, grupos en riesgo, grupos vulnerables, grupos en situación de vulnerabilidad, sistemas de conocimiento, redes de conocimiento, conocimiento indígena, conocimiento local, conocimiento territorial, saberes intergeneracionales, conocimiento ancestral, conocimientos tradicionales, rituales, tecnologías locales, reconocimiento, sistemas de creencias de los antiguos pueblos indígenas, ecosistemas más vulnerables, inclusión, inclusivas, agencia, desigualdades, desigualdades históricas, desigualdades estructurales/ desigualdades regionales/ factores estructurales de vulnerabilidad, distribución de los beneficios de la adaptación, zonas de riesgo/ zona vulnerable, patrones de la población, condiciones socioeconómicas, zonas más afectadas, mapas de riesgo, pobre, los más pobres, pobreza, pobreza extrema, marginados, prioridades de adaptación equitativa o justa, prioridades de adaptación, estrategias de adaptación territorial, capacidades institucionales de adaptación, capacidad adaptativa, grupos marginados, poblaciones excluidas, grupos étnicos, pueblos indígenas, migrantes, brechas de género, perspectiva de género, discriminación por género, grupos de atención prioritaria, coproducción de conocimiento, interculturalidad, educación intercultural. |
| **Procedural justice** | **Justicia procedimental** |
| differential participation**,** differential power**,** inclusive processes of representation, inclusive processes of participation**,** legitimacy**,** multiple stakeholders**,** deliberative dialogues**,** unequal power relations**.** | participación diferencial, poder diferencial**,** procesos inclusivos de representación**,** procesos inclusivos de participación**,** legitimidad**,** múltiples partes/contrapartes interesadas**,** diálogos deliberativos**,** relaciones de poder desiguales. |
| **Distributive justice** | **Justicia distributiva** |
| vulnerable groups, communities**,** structural vulnerabilities**,** distribution of benefits and burdens**,** fairness, fairly, equity, equitable**,** women, gender**,** responsiveness with climate services**,** adaptation funding for the poor and vulnerable social groups, prioritarianism**,** intergenerational equity | grupos, comunidades vulnerables**,** vulnerabilidades estructurales**,** distribución de beneficios y cargas**,** justicia, justamente**,** equidad/ equitativa/ equitativamente**,** mujer/género**,** capacidad de respuesta con los servicios climáticos**,** financiación para la adaptación de los grupos sociales pobres y vulnerables, prioritarismo, equidad intergeneracional. |

**Table 1.** This dataset provides a comprehensive list of keywords related to justice enablers - recognition, procedural and distributive justice - used in the analysis of National Adaptation Plans (NAPs), Health National Adaptation Plans (HNAPs) and their supporting documents. The keywords are categorised into three dimensions of justice and are provided in both English and Spanish to reflect the linguistic diversity of South America.

The dataset is a valuable resource for researchers and policy makers analysing the integration of justice considerations in climate adaptation planning. It provides a standardised set of terms that can be used to track and assess the presence of justice enablers in policy documents across countries and regions.

**Table 2**

List of NAPs, HNAPs, SASs

| **Country** | **Type of Document** | **Document** | **Date** | **Update Process** | **UNCCC** |
| --- | --- | --- | --- | --- | --- |
| **Argentina** | **National Adaptation Plan** | ‘Plan Nacional de Adaptación y Mitigación al Cambio Climático’ | 2022 | not identifiable | not submitted |
|  | ***Subsidiary Document with a National Adaptation Strategy*** | - | - | - | - |
|  | **Health National Adaptation Plan** | ‘Estrategia Nacional de Salud y Cambio Climático’ | 2023 | not identifiable | not submitted |
|  | ***Subsidiary Document with a National Health Adaptation Strategy*** | - | - | - | - |
| **Bolivia** | **National Adaptation Plan** | - | - | - | - |
|  | ***Subsidiary Document with a National Adaptation Strategy*** | ‘Política Plurinacional de Cambio Climático’ | 2016 | - | - |
|  | **Health National Adaptation Plan** |  | - | - | - |
|  | ***Subsidiary Document with a National Health Adaptation Strategy*** | ‘Cambio Climático y el Desafío de la Salud en Bolivia’ | 2013 | - | - |
| **Brazil** | **National Adaptation Plan** | ‘National Adaptation Plan to Climate Change (Vol. I): General Strategy’ | 2016 | not identifiable | submitted 2016 |
|  | ***Subsidiary Document with a National Adaptation Strategy*** | - | - | - | - |
|  | **Health National Adaptation Plan** | ‘National Adaptation Plan to Climate Change (Vol: 2): Sectoral and Thematic Strategies’ | 2016 | not identifiable | submitted 2016 |
|  | ***Subsidiary Document with a National Health Adaptation Strategy*** | - | - | - | - |
| **Chile** | **National Adaptation Plan** | ‘Plan Nacional de Adaptación al Cambio Climático’ | 2014 | ongoing | submitted 2017 |
|  | ***Subsidiary Document with a National Adaptation Strategy*** | - | - | - | - |
|  | **Health National Adaptation Plan** | ‘Plan de Adaptación al Cambio Climático para Salud’ | 2017 | not identifiable | not submitted |
|  | ***Subsidiary Document with a National Health Adaptation Strategy*** | - | - | - | - |
| **Colombia** | **National Adaptation Plan** | ‘Plan Nacional de Adaptación al Cambio Climático’ | 2016 | not identifiable | submitted 2018 |
|  | ***Subsidiary Document with a National Adaptation Strategy*** | - | - | - | - |
|  | **Health National Adaptation Plan** | - | - | - | - |
|  | ***Subsidiary Document with a National Health Adaptation Strategy*** | ‘Plan Decenal Salud Pública 2022–2031’ | 2022 | not identifiable |  |
| **Ecuador** | **National Adaptation Plan** | ‘Plan Nacional de Adaptación al Cambio Climático del Ecuador 2023–2027’ | 2023 | not identifiable | not submitted |
|  | ***Subsidiary Document with a National Adaptation Strategy*** | - | - | - | - |
|  | **Health National Adaptation Plan** | - | - | - | - |
|  | ***Subsidiary Document with a National Health Adaptation Strategy*** | ‘Plan Decenal de Salud 2022–2031’ | 2022 | - | - |
| **Guyana** | **National Adaptation Plan** | - | - | - | - |
|  | ***Subsidiary Document with a National Adaptation Strategy*** | ‘National Drought Mitigation and Adaptation Plan’ | 2020 | - | - |
|  | **Health National Adaptation Plan** | - | - | - | - |
|  | ***Subsidiary Document with a National Health Adaptation Strategy*** | ‘Health and Climate Change—Country Profile 2020: Guyana’ | 2020 | - | - |
| **Paraguay** | **National Adaptation Plan** | ‘Plan Nacional de Adaptación al Cambio Climático 2022–2030’ | 2022 | not identifiable | submitted 2022 |
|  | ***Subsidiary Document with a National Adaptation Strategy*** | - | - | - | - |
|  | **Health National Adaptation Plan** | - | - | - | - |
|  | ***Subsidiary Document with a National Health Adaptation Strategy*** | ‘Política Nacional de Salud 2015–2030’ | 2015 | - | - |
| **Peru** | **National Adaptation Plan** | ‘Plan Nacional de Adaptación al Cambio Climático del Perú: Un Insumo para la Actualización de la Estrategia Nacional ante el Cambio Climático’ | 2021 | not identifiable | submitted 2021 |
|  | ***Subsidiary Document with a National Adaptation Strategy*** | - | - | - | - |
|  | **Health National Adaptation Plan** | - | - | - | - |
|  | ***Subsidiary Document with a National Health Adaptation Strategy*** | ‘Programación Tentativa de las Contribuciones Nacionalmente Determinadas en Adaptación frente al Cambio Climático del Ministerio de Salud al 2030’ | 2022 | not identifiable | - |
| **Suriname** | **National Adaptation Plan** | ‘Suriname National Adaptation Plan 2019–2029’ | 2019 | not identifiable | submitted 2020 |
|  | ***Subsidiary Document with a National Adaptation Strategy*** | - | - | - | - |
|  | **Health National Adaptation Plan** | ‘Suriname National Adaptation Plan 2019–2029’ | 2019 | not identifiable | submitted 2020 |
|  | ***Subsidiary Document with a National Health Adaptation Strategy*** | - | - | - | - |
| **Uruguay** | **National Adaptation Plan** | - | - | - | - |
|  | ***Subsidiary Document with a National Adaptation Strategy*** | ‘Plan Nacional de Adaptación a la Variabilidad y el Cambio Climático para el Sector Agropecuario de Uruguay’ | 2019 | not identifiable | submitted 2019 |
|  |  | ‘Plan Nacional de Adaptación para la Zona Costera’ | 2021 | not identifiable | submitted 2021 |
|  |  | ‘Plan Nacional de Adaptación al Cambio Climático en Ciudades e Infraestructuras-NAP Ciudades’ | 2021 | not identifiable | submitted 2021 |
|  | **Health National Adaptation Plan** | - | - | - | - |
|  | ***Subsidiary Document with a National Health Adaptation Strategy*** | - | - | - | - |
| **Venezuela** | **National Adaptation Plan** | - | - | - | - |
|  | ***Subsidiary Document with a National Adaptation Strategy*** | ‘Actualización de la Contribución Nacionalmente Determinada de la República Bolivariana de Venezuela para la Lucha contra el Cambio Climático y sus Efectos’ | 2021 | not identifiable | - |
|  | **Health National Adaptation Plan** | - | - | - | - |
|  | ***Subsidiary Document with a National Health Adaptation Strategy*** | - | - | - | - |

**Table 2.** This dataset provides a detailed overview of the National Adaptation Plans (NAPs), Health National Adaptation Plans (HNAPs) and Subsidiary Adaptation Strategies (SASs) of several South American countries. The table includes information on the type of document (e.g., National Adaptation Plan, Health National Adaptation Plan, Subsidiary Strategy), the specific document name, the date of publication and the status of submission to the UNFCCC portal.

The dataset covers countries such as Argentina, Bolivia, Brazil, Chile, Colombia, Ecuador, Guyana, Paraguay, Peru, Suriname, Uruguay and Venezuela, highlighting the diversity of climate adaptation planning across the region. For each country, the dataset indicates whether the document has been submitted to the UNFCCC portal. This comprehensive dataset is a valuable resource for researchers and policymakers analysing South America's progress and challenges in climate adaptation planning.

**Table 3**

Integration of justice enablers in NAPs, HNAPs, and SASs

|  | **NAPs/Subsidiary Documents** | | | **HNAPs/Subsidiary Documents** | | |  |
| --- | --- | --- | --- | --- | --- | --- | --- |
|  | Recognitional Justice | Procedural Justice | Distributive Justice | Recognitional Justice | Procedural Justice | Distributive Justice | |
| **Argentina** | 72 | 156 | 77 | 19 | 23 | 9 | |
| **Bolivia** | 4 | 1 | 34 | 26 | 44 | 10 | |
| **Brazil** | 18 | 25 | 16 | 8 | 7 | 4 | |
| **Chile** | 33 | 49 | 3 | 19 | 10 | 12 | |
| **Colombia** | 45 | 66 | 4 | 37 | **217** | 7 | |
| **Ecuador** | 34 | 53 | 7 | **41** | 100 | **23** | |
| **Guyana** | NA* | NA | NA | 6 | 0 | 0 | |
| **Paraguay** | 33 | 41 | 12 | 7 | 13 | 6 | |
| **Perú** | 90 | 91 | 37 | 19 | 8 | 15 | |
| **Suriname** | 69 | 112 | 9 | 4 | 6 | 0 | |
| **Uruguay** | **114** | **547** | **92** | NA | NA | NA | |
| **Venezuela** | 77 | 81 | 26 | NA | NA | NA | |

* NA: Document not found

The leading case that marks the reference threshold is highlighted in bold

|  | **NAPs/Subsidiary Documents** | | | | **HNAPs/Subsidiary Documents** | | |
| --- | --- | --- | --- | --- | --- | --- | --- |
|  | Recognitional Justice | Procedural Justice | Distributive Justice | Recognitional Justice | | Procedural Justice | Distributive Justice |
| **low** | < 38 | < 82 | < 31 | < 14 | | < 72 | < 8 |
| **medium** | ≥ 38 and < 76 | ≥ 182 and < 364 | ≥ 31 and < 62 | ≥ 14 and < 28 | | ≥ 72 and < 144 | ≥ 8 and < 16 |
| **high** | ≥ 76 | ≥ 364 | ≥ 62 | ≥ 28 | | ≥ 144 | ≥ 16 |

**Table 3**. This dataset provides a detailed assessment of justice enablers - recognitional, procedural and distributive justice - in the National Adaptation Plans (NAPs), Health National Adaptation Plans (HNAPs) and their subsidiary documents in South American countries. The table categorises the integration of justice dimensions into low, medium and high levels based on predefined thresholds. The dataset includes quantitative scores for each justice dimension (recognitional, procedural, distributive) in both NAPs/subsidiary documents and HNAPs/subsidiary documents.

The dataset covers 12 South American countries, some of which have no HNAPs or subsidiary documents (marked as 'NA'). The thresholds for classifying the enabling potential of the justice dimensions are as follows

- Low: Scores below the lower threshold.

- Medium: Scores between the lower and upper thresholds.

- High: scores at or above the upper threshold.

For example, Uruguay's NAPs show high scores for recognition and procedural justice, while Bolivia's NAPs show low scores for procedural justice. The dataset also highlights disparities in the integration of justice, with some countries excelling in recognitional justice but lagging behind in distributive justice. This dataset is a valuable resource for researchers and policymakers analysing equity and justice in climate-health adaptation planning across South America.

**Experimental Design, Materials and Methods**

Data were collected from publicly available National Adaptation Plans (NAPs), Health National Adaptation Plans (HNAPs), and Subsidiary Adaptation Strategies (SASs) from 12 South American countries. Justice enablers were identified using keyword-based analysis, with keywords translated into Spanish and Portuguese to reflect regional linguistic diversity. The enabling potential of each justice dimension was classified as low, medium or high based on its integration and relevance in the actionable sections of the plans. Statistical analysis was performed to identify trends and differences in justice inclusion across countries and plan types.

**Ethics Statement**

None.

**Declaration of Competing Interest**

The authors declare that they have no known competing financial interests or personal relationships that could have appeared to influence the work reported in this paper.

**Data Availability**

Equity Enablers of Climate-Health Adaptation in National Adaptation Plans in South America. Available at <https://osf.io/f42ar/files/osfstorage> <https://doi.org/10.17605/OSF.IO/DP65>
